# Synthetic Advances in Macrosphelides: Natural Anticancer Agents

**DOI:** 10.3390/molecules191015982

**Published:** 2014-10-08

**Authors:** Seung-Mann Paek

**Affiliations:** College of Pharmacy and Research Institute of Pharmaceutical Sciences, Gyeongsang National University, Jinju daero, Jinju, Gyeongnam 660-701, Korea

**Keywords:** macrosphelides, total synthesis

## Abstract

Total synthesis of macrosphelides is summarized. Synthetic approaches contain the preparation of key fragments and the final ring-closure reaction for unique 16- or 15-membered macrolactone skeletons.

## 1. Introduction

**Figure 1:**
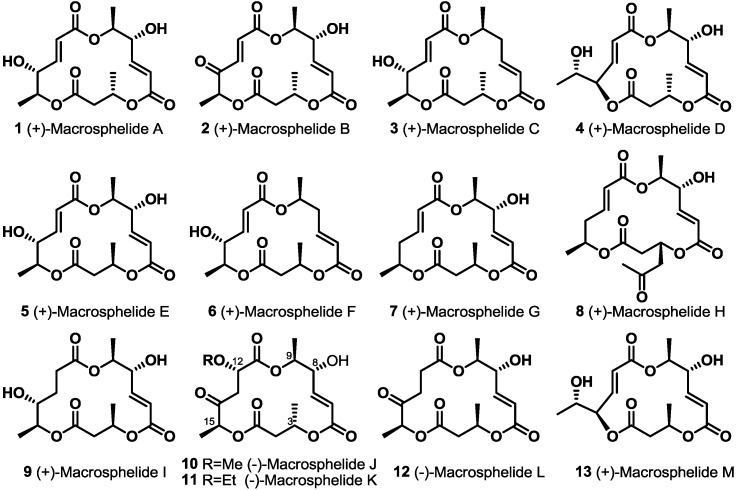
Natural macrosphelides (MSs).

Macrosphelides (MS), natural macrolide polyketides, were first reported as new anticancer metastasis agents in 1995 [1]. Because this new macrolide showed promising biological activities, such as inhibition of the adhesion of HL60 cells to human umbilical-vein endothelial cells (HUVEC) [2,3,4,5,6,7], no acute toxicity by i.p*.* injection of MSA into BDF1 mice at 200 mg/kg for five days [8], potent immunosuppressant activity *in vivo* [9], anticancer activity against lung metastasis of B16/BL6 melanoma in mice [10] and antimicrobial activity [11], significant endeavors to synthesize this natural product family and isolate-related MS isotypes have been carried out [12,13]. So far, 13 natural isomers have been reported from *Microsphaeropsis* sp. FO-5050, *Periconia byssoides*, *Coniothyrium minitans* and the fungus, *Tritirachium* sp*.* HKI 0317 [14]. In addition, about 20 synthetic approaches have been developed for more efficient preparation. Herewith, synthetic advances in natural MS are summarized (Figure 1).

## 2. Results and Discussion

### 2.1. Synthetic Features of MS

**Figure 2:**
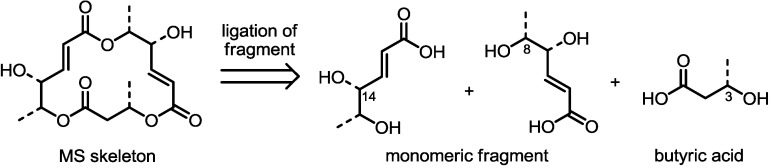
Synthetic features of MS preparation.

As shown in Figure 2, MS usually possesses a 16-membered macrolactone core with three ester linkages. In the retrosynthetic view, this skeleton can be divided into three hydroxy acids. It is important to note that two of them feature an almost identical carbon framework and oxidation state. This means that just one efficient synthesis of the fragment may complete full synthesis of MS itself after appropriate protection group modifications and ligation of the fragments. Actually, most methods of MS synthesis involve a unique preparation method of the monomeric fragment. For the final macrocyclization reaction, the Yamaguchi macrolactonization reaction [15] was chosen as the first option. Recently, however, other useful C-C bond formations have been developed to create special 16-membered macrolactones efficiently. These variations in the synthetic procedure make it possible to prepare a variety of MS-related compounds and improve their activities.

### 2.2. The First Total Synthesis of MSA and MSB

**Scheme 1:**
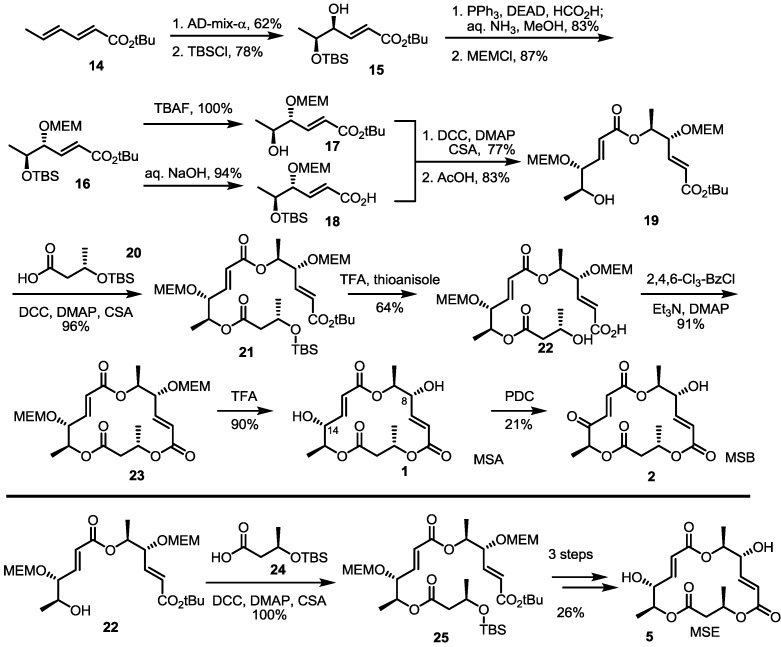
The first total synthesis of MSA, B and E.

As the main isomers of the MS family, the first synthetic approach was developed through a collaboration of the Smith and Omura groups, as shown in Scheme 1 [8]. This first synthesis contains not only the synthetic route to MSA/B, but also a determination of the absolute structures of these natural products.

MEM: 2-Methoxyethoxymethyl, TBS: tert-Butyldimethylsilyl, DCC: Dicyclohexylcarbodiimide, TBAF: Tetrabutylammonium Fluoride, TFA: Trifluoro aceticacid, DMAP: 4-(Dimethylamino)pyridine, CSA: Camphor-10-sulfonic acid, DEAD: Diethyl azodicarboxylate, PDC: Pyridinium dichromate.

Asymmetric dihydroxylation of t-butyl sorbate and chemoselective silyl protection produced allylic alcohol **15** in a good yield. Mitsunobu inversion of the allylic alcohol configuration and MEM protection produced bis-protected diol **16**, which could be used as a common intermediate for further transformation. Silyl deprotected alcohol **17** and hydrolyzed carboxylic acid **18** were coupled to produce a dimerized ester that was converted into secondary alcohol **19** using AcOH in 83% yield. Esterification of **19** with butyric acid **20** under Keck conditions [16], followed by acidic silyl deprotection and concomitant t-butyl hydrolysis, gave the crucial seco acid in moderation. Finally, Yamaguchi macrolactonization and MEM deprotection produced MSA **1** in an excellent yield. MSA could be oxidized to MSB **2** using PDC (Pyridinium dichromate) reagent. Although this final oxidation produced a C-8 oxidized product in high yield, it is important that this conversion first confirmed the absolute structure of MSB.

Eight years later, total synthesis of MSE was also reported by employing a similar reaction sequence [17]. Instead of **20**, **24** was coupled with the secondary hydroxyl ether **25**, which was transformed into MSE **5** via the same conversion protocol as MSA.

### 2.3. Synthesis of MSA and MSB by Furan Oxidation

**Scheme 2:**
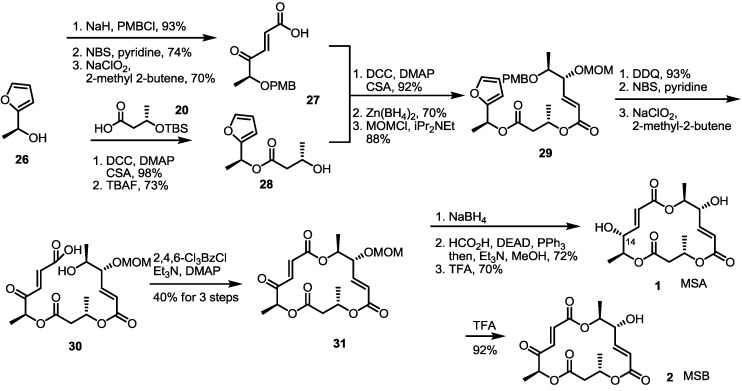
Total synthesis of MSA and MSB through furan oxidation.

The second total synthesis of MS was accomplished using the furan oxidation strategy [18,19]. The Kobayashi group reported conversion of furan to γ-keto-α,β-unsaturated carboxylic acid using NBS, and then Pinnick oxidation [20] could be used for efficient preparation of the MS skeleton framework (Scheme 2). Actually, versatile conversion of the hydroxyfuran **26** produced not only carboxylic acid **27** through the PMB (p-Methoxybenzyl) protection/furan oxidation sequence, but also secondary alcohol **28** through the esterification/silyl deprotection sequence, respectively. They were connected together under Keck conditions to give a dimeric ester, which was reduced, and the resulting hydroxyl group was protected with the MOM (Methoxymethyl) group to form **29**. It was possible to create new stereogenic centers in high selectivity (>15:1) using Zn(BH_4_)_2_, −90 °C and the reverse addition strategy. To produce seco acid **30**, PMB deprotection/furan oxidation of dimer **29** was carried out successfully. The 16-memberd macro-ring closure reaction under Yamaguchi conditions produced MOM-protected MSB **31**. Unlike MSB, which was synthesized by simple acidic deprotection (92%), however, MSA was difficult to produce, because the reduction of the ketone moiety gave the undesired C14 epimer exclusively. Finally, Mitsunobu inversion of the C14 hydroxy group and deprotection were executed to create MSA from the MOM-protected MSB **31**.

PMB: p-Methoxybenzyl, NBS: *N*-Bromosuccinimide, DDQ: 2,3-Dichloro-5,6-dicyanobenzoquinone, MOM: Methoxymethyl.

**Scheme 3:**
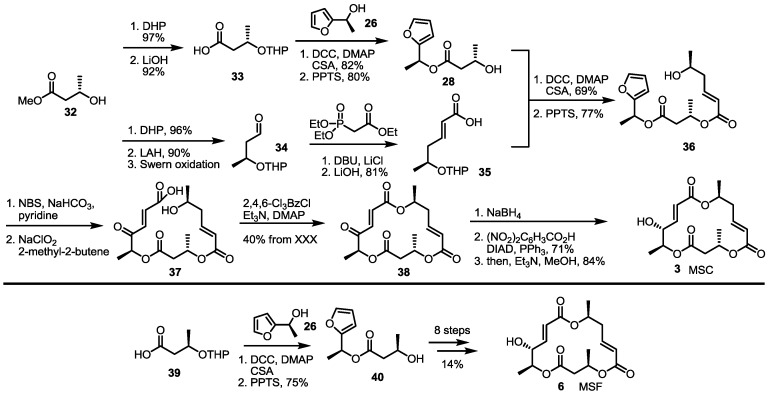
Total synthesis of MSC and MSF through furan oxidation.

Employing the late-stage furan oxidation protocol, 8-dehydroxy MS was prepared [21] (Scheme 3). The hydroxy butyrate **32** was protected and hydrolyzed to produce butyric acid **33** to be converted into hydroxyfuran **28** through esterification and THP deprotection. This alcohol **28** was transformed into dimeric alcohol **36** via esterification/deprotection with carboxylic acid **35**, which were prepared by Horner-Emmons olefination and hydrolysis of the known aldehyde **34** [22]. The furan moiety of alcohol **36** was oxidized with NBS and the Pinnick oxidation sequence to produce seco acid **37**. The well-established Yamaguchi lactonization, reduction and the Mitsunobu reaction [23] produced MSC in a good yield. Instead of butyric acid **33**, enantiomeric acid **39** was utilized to give MSF possessing a C-13 epimeric chiral center of MSC. The established procedure made it possible to produce MSF in 10 steps.

DHP: Dihydropyran, LAH: Lithium aluminium hydride, PPTS: Pyridinium p-toluenesulfonate, DBU: 1,8-Diazabicyclo[5.4.0]undec-7-ene, DIAD: Diisopropyl azodicarboxylate.

**Scheme 4:**
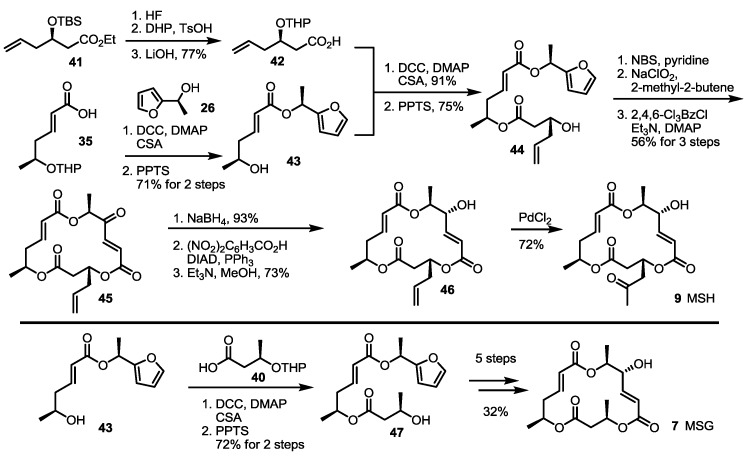
Total synthesis of MSH and MSG through furan oxidation.

The Kobayashi group also reported total synthesis of MSH/G in 2002 [24]. The known hexenoate **41** [25] was converted into carboxylic acid **42** through protection group exchange and hydrolysis. This carboxylic acid was esterified with the furan **43**, prepared from the previously synthesized **35** via esterification, followed by THP deprotection, to produce the alcohol **44**, after treatment with PPTS, in a 75% yield. Crucial NBS-mediated furan oxidation/Pinnick oxidation/Yamaguchi lactonization gave trimeric ester **45** in 56% yield. After introduction of the C-14 hydroxy group, the terminal alkene was oxidized to a ketone through Wacker oxidation to form MSH **9**. Instead of **42** as its esterification partner, simple carboxylic acid **39** was chosen to produce MSG **7**, employing the well-established seven steps shown in Scheme 4.

THP: Tetrahydropyran, HF: Hydrogen fluoride.

### 2.4. Synthesis of MS by Enzymatic Resolution

**Scheme 5:**
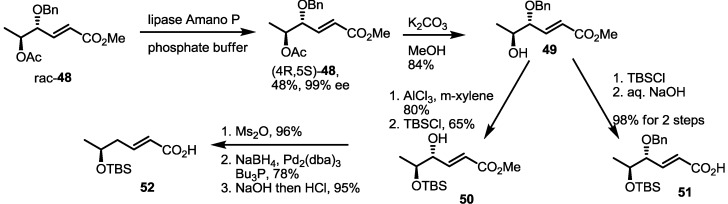
Chiral resolution of racemic **48** and preparation of building blocks.

Enzymatic resolution of racemic **48** was also developed for MS synthesis [26,27] (Scheme 5). Acetylated anti-diol **48** was differentiated under catalytic hydrolysis of lipase Amano P to produce optically active (4*R*,5*S*)-**48** in an excellent selectivity. Deacetylation of (4*R*,5*S*)-**48** produced versatile synthon **49**. Actually, the TBS protection/hydrolysis protocol gave the key monomeric acid **51**, used for general synthesis of MS. At the same time, protection group exchange of **49** was carried out to prepare **50**. Then, deoxygenation was executed via mesylation followed by Pd-mediated hydride insertion [28]. After hydrolysis of the methyl ester, monohydroxy carboxylic acid **52** was obtained in a good yield and utilized for the synthesis of deoxygenated MSs.

Ac: Acetyl, Bn: Benzyl.

**Scheme 6:**
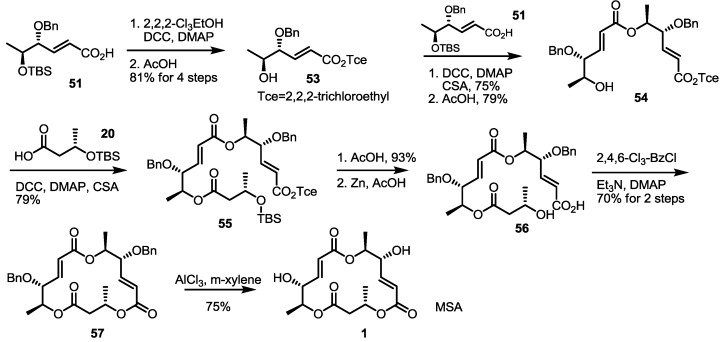
Total synthesis of MSA and MSE through an enzymatic resolution route.

Employing these building blocks, MS synthesis was carried out (Scheme 6). Carboxylic acid **51** was transformed into **53** via trichloroethyl esterification and acidic deprotection. Iterative esterification of **53** with acid **51**, deprotection of the TBS group, esterification with acid **20**, deprotection of the TBS group and treatment with zinc produced seco acid **56** in a good yield. Finally, macrolactonization and benzyl deprotection gave MSA in a good yield.

**Scheme 7:**
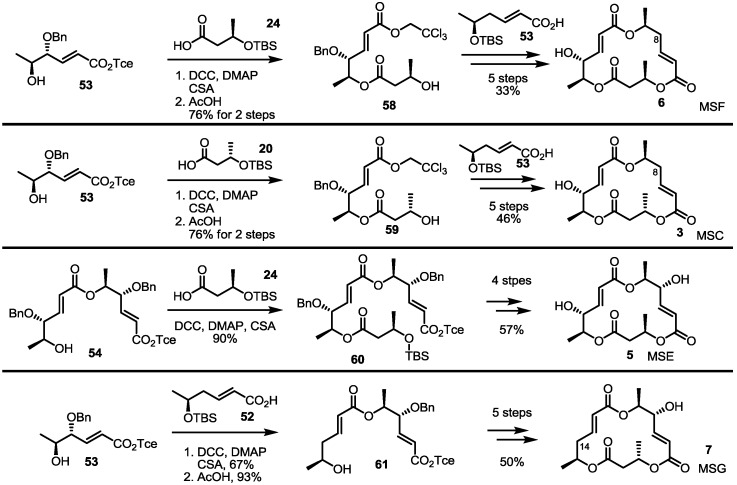
Total synthesis of MSE, G, F and C.

Similarly, MSE was also prepared by esterification with **20** and butyric acid **24** and additional transformations (four steps, 57% yield) (Scheme 7). Deoxygenated MSs was also prepared using this synthetic protocol and building blocks. Esterification of **53** with deoxygenated carboxylic acid **52** and deprotection gave dimer **61** in a good yield. The established 5-step procedure produced MSG. Coupling of butyric acid **20**/**24** with alcohol **53** produced **59** and **58**, depending on the chiral center of butyric acid [29]. They were successively converted into MSF/C through normal transformations.

**Scheme 8:**
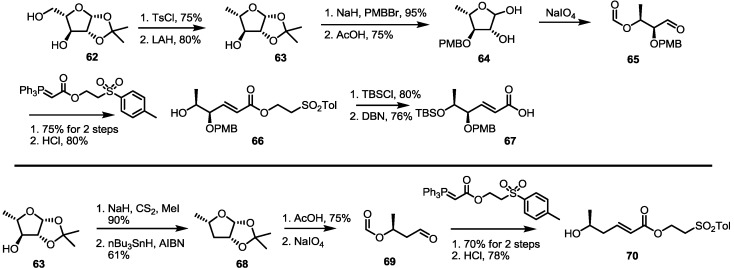
Preparation of building blocks from sugar species.

Another approach that used sugar building blocks was also developed for MS synthesis by the Sharma group [30,31]. To prepare the 1,2-antihydroxy carboxylic acid moiety, pentose **62** was tosylated and reduced, as shown in Scheme 8. PMB protection and deacetalization, followed by the diol cleavage reaction, gave unstable aldehyde **65**. Immediate Wittig olefination and deformylation produced the desired bis-hydroxy ester **66** easily. TBS protection and de-esterification under basic conditions also gave another building block, **67**. Deoxygenated sugar **63** was also utilized to prepare mono-hydroxy ester **70**. Xanthate formation and radical-mediated deoxygenation produced deoxy sugar **68** in a good yield. After application of the previously established four-step procedure, mono-hydroxy ester **70** was quickly prepared.

AIBN: Azobisisobutyronitrile, DBN: 1,5-Diazabicyclo[4.3.0]non-5-ene.

**Scheme 9:**
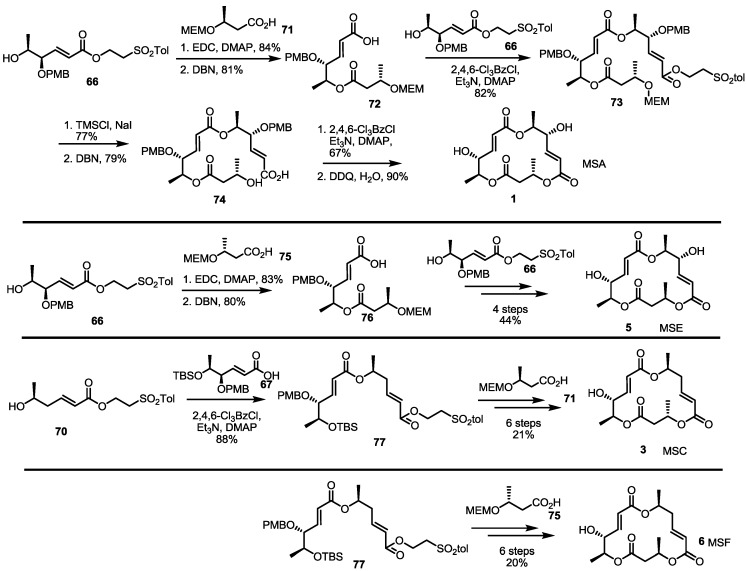
Total synthesis of MSA, E, C and F using building blocks from sugar species.

MS synthesis using sugar synthon is summarized in Scheme 9. Hydroxy ester **66** was coupled with protected butyric ester and treated with DBN to give dimeric acid **73**. Again, esterification with hydroxyl ester **66** produced the fully substituted ester **73** in an 82% yield. Macrolactonization, deprotection and de-esterification were executed to produce seco acid **74**, which was converted into MSA **1** after Yamaguchi lactonization and the PMB deprotection sequence. After establishing the synthetic protocol, MS isomers E, C and F were also synthesized. Instead of **71**, enantiomeric acid **75** was utilized in the synthesis of MSE **5**. Actually, the normal deprotection/esterification protocol produced MSE **5**. For deoxygenated isomer MSC/F, deoxygenated fragment **70** was used. Esterification of **70** with carboxylic acid **67** produced a versatile intermediate **77** in an excellent yield. Employing butyric acid **71** or **75**, MSC **3** or MSF **6** was synthesized in six steps, depending on the chirality of the coupling partner.

EDC: *N*-(3-Dimethylaminopropyl)-*N*′-ethylcarbodiimide hydrochloride.

**Scheme 10:**
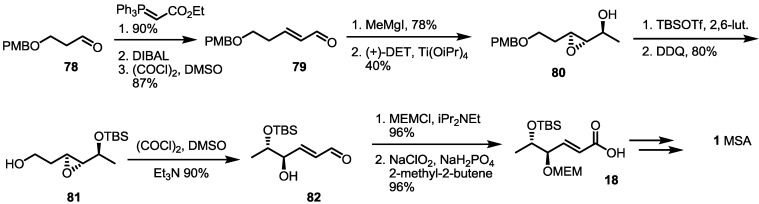
Formal synthesis of MSA using an oxidation/elimination protocol.

Another synthetic approach using chiral resolution was reported in 2003 [32] (Scheme 10). A simply protected butanal **78** was treated with Wittig reagent, followed by DIBAL (Diisobutyl aluminium hydride) reduction/Swern oxidation to produce hexenal **79** in an excellent yield. After Grignard reaction with MeMgI, the resulting allylic alcohol was exposed to Sharpless asymmetric epoxidation for chiral resolution (94% enantiomeric excess). An optically active alcohol **80** was then protected with TBS, and primary alcohol was liberated with the help of DDQ to give epoxy alcohol **81**. Free alcohol **81** was then oxidized under Swern oxidation conditions, and concomitant aldehyde was spontaneously transformed into the desired hexenal **82** via beta-elimination. This aldehyde was protected and oxidized to produce the known carboxylic acid **18** in an excellent yield [8].

DET: Diethyl tartrate, DIBAL: Diisobutyl aluminium hydride.

**Scheme 11:**
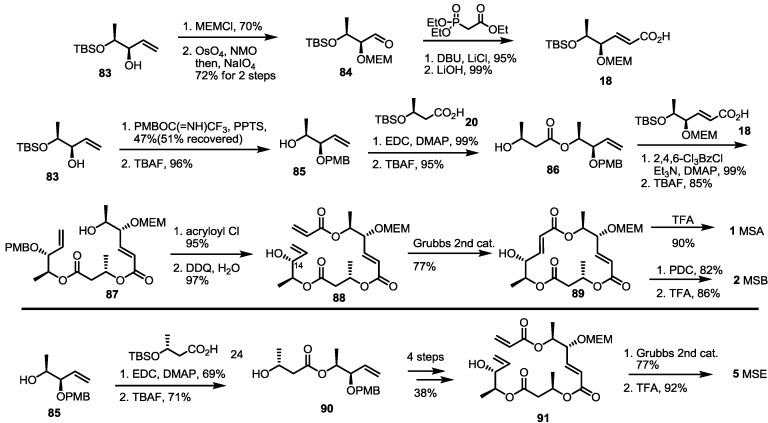
Total synthesis of MSA, B and E by the ring-closing metathesis (RCM) strategy.

Instead of the Yamaguchi lactonization reaction as macrocyclization, another strategy has been studied since 2003. For this purpose, ring-closing metathesis (RCM) was applied to synthesize the MS core structure successfully, as shown in Scheme 11 [33]. The Nemoto group reported that the known allyl alcohol **83** could be converted into MS using this strategy. Protection of the allyl alcohol **83** with the MEM group and oxidative cleavage produced bis-protected aldehyde **84**, which can be homologated using Horner–Emmons reagent to produce hexenoic acid **18** after basic hydrolysis. Allyl alcohol **83** was also protected with the PMB group, and TBS was eliminated to prepare homoallylic alcohol **85**. Esterification with butyric acid **20** and iterative TBS deprotection gave hydroxyl ester **86** in an excellent yield. Again, esterification with acid **18** and deprotection gave the secondary alcohol **87**, which was converted into pivotal acrylate **88** after acryloylation and PMB deprotection. After an extensive substrate and reaction conditions survey, the RCM reaction was carried out sufficiently to give the macrolactone skeleton **89** using Grubbs second catalyst and refluxing CH_2_Cl_2_. It is important to note that the RCM did not work well in the presence of the PMB group, due to the C-14 protection group. This discrepancy means that insertion of the substrate into the ruthenium core depends on the steric factor of the substrate. Except for Yamaguchi cyclization, the RCM strategy was the first protocol to create an MS macrolactone core. In addition, the RCM strategy was applied to the synthesis of other MS and MS artifacts, because of its efficiency and easy preparation of the corresponding substrates, as will be shown later (Scheme 16). Finally, MSA **1** was prepared from the macrolactone **89** after acidic deprotection, while MSB **2** was prepared using oxidation/deprotection reactions. In addition, MSE **5** was synthesized from homoallylic alcohol **85** using butyric acid **24** instead of **20** after a similar reaction protocol [34].

**Scheme 12:**
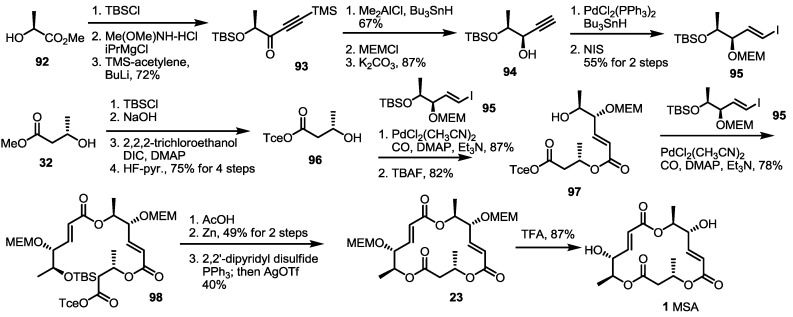
Total synthesis of MSA using the CO insertion strategy by the Takahashi group.

Pd-mediated CO insertion was also developed for MS synthesis by the Takahashi group [35] (Scheme 12). Methyl lactate **92** was transformed into alkynone **93** by TBS protection, Weinreb amide formation and alkynylation. After selective reduction of the carbonyl group to a hydroxyl group, free alcohol was protected using MEM to give propargylic alcohol **94** after TMS deprotection. This alkyne was then converted into vinyl iodide **95** via a Pd-catalyzed reaction and NIS (*N*-Iodosuccinimide) treatment. The vinyl iodide **95** was coupled with hydroxyl butyrate **96**, prepared from methyl ester **32** and the ester exchange reaction, to produce dimer **97** after TBS deprotection. This C-C and C-O bond formation reaction with CO insertion into the vinyl iodide produced the secondary alcohol in an excellent yield. The same reaction was utilized again to give trimeric ester **98** in 78% yield. After TBS/trichloroethyl deprotection, the corresponding seco acid was cyclized to the bis-protected macrolactone **23** [8] under a modified Mukaiyama–Corey lactonization condition in a moderate yield. It is noteworthy that the addition of AgOTf was crucial to perform cyclization reaction. Finally, acidic deprotection produced MSA **1** in a good yield. This Pd(0)-mediated carbonylative insertion strategy also utilized the combinatorial chemistry of MS to prepare the MS-related chemical library successfully [36].

NIS: *N*-Iodosuccinimide, DIC: *N*,*N*′-Diisopropylcarbodiimide.

A vinylogous ester anion was found to be a useful synthon for the MS skeleton in 2005 [37]. Protected lactic acid **99** was converted into Weinreb amide **100** (90%), which was treated with the lithiated vinylogous orthoester anion to give unsaturated ketone **102** in a 90% yield [38,39]. After chelation-controlled reduction, MEM protection and hydrolysis, monomeric acid **103** was obtained quickly. This building block was esterified and deprotected to homoallyl ester **104**, which was transformed into dimeric ester **105** via iterative esterification/deprotection reactions. Finally, esterification, PMB deprotection and allyl deprotection produced the known seco acid **22** in an excellent yield [8].

**Scheme 13:**
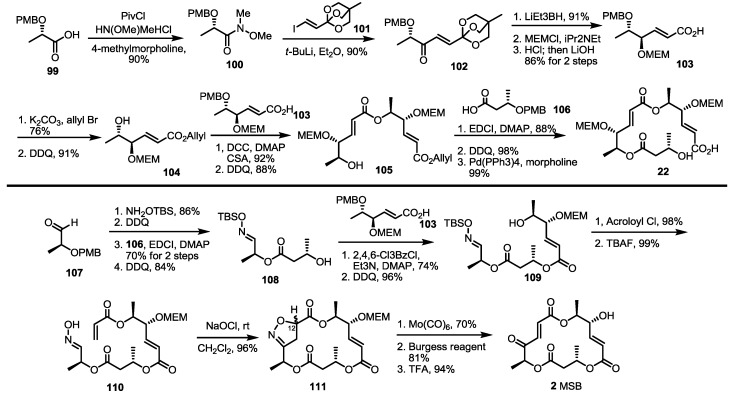
Synthesis of MSA and MSB using orthoester addition and cycloaddition.

**Scheme 14:**
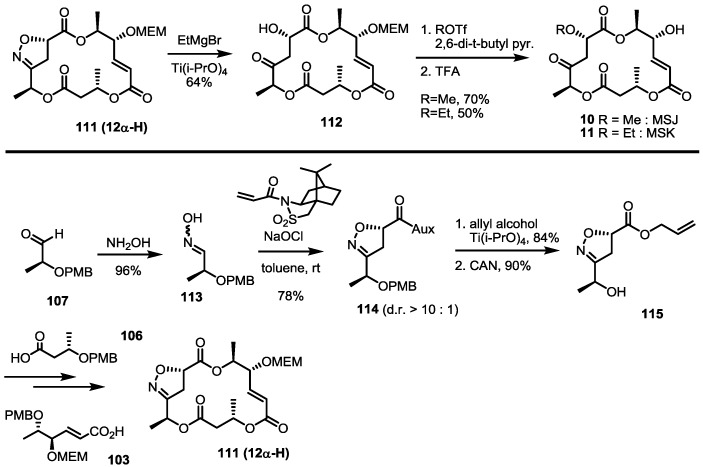
Structural determination of MSJ and MSK through total synthesis.

A more important advance was the finding that nitrile oxide cycloaddition could be used to construct the MS skeleton, generating an unstable ketone moiety at the same time (Scheme 13). The known aldehyde **107** was converted into alcohol **108** using oxime formation, esterification and deprotection [40]. This alcohol was also coupled with carboxylic acid **103** to give dimeric alcohol **109** after PMB deprotection. Acryloylation and TBS deprotection produced the desired oxime **110** quickly. Pivotal intramolecular nitrile oxide cycloaddition was carried out at this stage to prepare isoxazoline **111**, which possesses latent unsaturated ketone functionality. Fortunately, isoxazoline **111** was obtained in an excellent regioselectivity, while stereoselectivity varied depending on the reaction conditions, such as the solvent and additives [41,42]. Although C12 stereogenic center would be eliminated for MSB **2** synthesis, it was important for MSJ/K synthesis (Scheme 14). Reductive cleavage of the N-O bond, elimination of the secondary alcohol and acidic deprotection produced MSB **2** directly.

Tf: Trifluoromethanesulfonate, CAN: Ceric ammoniumnitrate.

After total synthesis of MSA and B was completed, the synthetic intermediate **111** was utilized to elucidate the structure of MSJ **10** and K **11** [43]. As expected, reductive cleavage of isoxazoline, O-alkylation and the acidic deprotection sequence produced the desired product, which possesses all spectral data matching natural MSJ and K, allowing confirmation of the exact structure of MSJ and K. Because the 12-α isoxazoline **111** was usually a minor product of the corresponding cycloaddition, auxiliary-assisted cycloaddition was also designed (Scheme 14). The free oxime **113** and acrylated sultam were ligated to give isoxazoline **114** in an excellent diastereoselectivity via intermolecular cycloaddition. Ester exchange and PMB deprotection produced allyl ester **115**, which was converted into isoxazoline **111** using the established esterification/deprotection sequence.

**Scheme 15:**
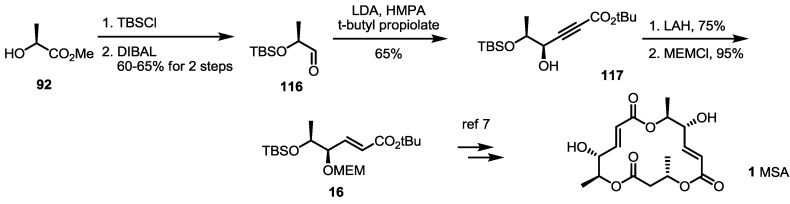
Formal synthesis of MSA using an alkyne reduction strategy.

As another formal synthesis method for MSA, an alkyne reduction strategy was also reported in 2005 [44] (Scheme 15). TBS-protected aldehyde **116** was prepared from methyl lactate **92** and TBS protection/semi-reduction. Alkyne addition under basic conditions was carried out to get propargylic alcohol **117**, which was reduced and protected to allylic ether **16** [8]. This known ether **16** could be used to synthesize MSA **1**, as reported previously.

LDA: Lithium diisopropylamide, HMPA: Hexamethylphosphoramide.

**Scheme 16:**
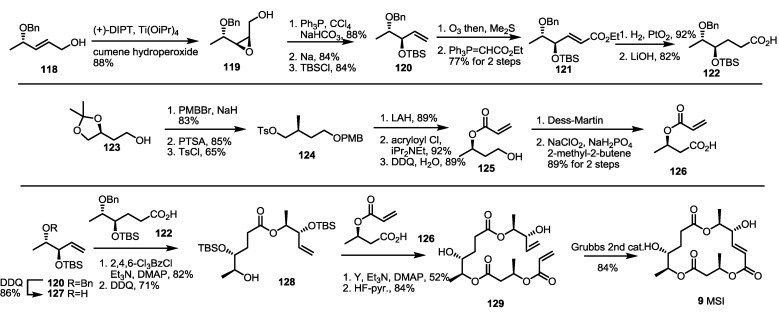
Total synthesis of MSI and dihydro-MSE.

A dihydro-MS core structure was synthesized in 2007 by the Sharma group [45] (Scheme 16). They used Sharpless asymmetric epoxidation of the known alcohol **118** to obtain epoxy alcohol **119**. The terminal hydroxyl group was chlorinated and eliminated with the assistance of Na to produce terminal alkene **120** after TBS protection. Ozonolysis and Wittig homologation produced hexenoate **121**, which was transformed to hexanoic acid **122** by hydrogenation and hydrolysis. At the same time, the known primary alcohol **123** was protected with the PMB group (83%) [46]. After deacetalization and tosylation of another primary alcohol, tosylate **124** was obtained in a good overall yield and converted into acrylate **125** through reduction/acryloylation/PMB deprotection. Two-step oxidation of the primary alcohol into carboxylic acid also produced another building block, **126**. Ligation of the three fragments commenced with debenzylation of ether **121** with DDQ. The corresponding alcohol **127** was esterified with carboxylic acid **128** to give dimeric alcohol after PMB deprotection. The second esterification with acrylic acid **126** was performed under the same conditions to give trimeric ester **129** after TBS deprotection again. Finally, RCM of the terminal diene **129** delivered MSI **9** in an excellent yield, as expected.

**Scheme 17:**
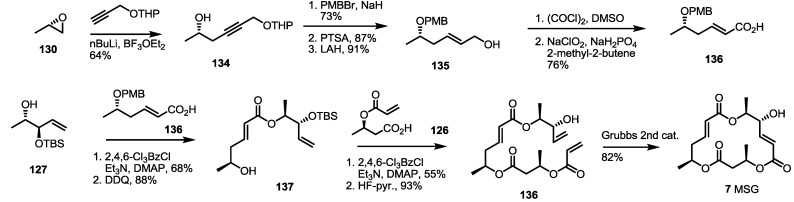
Total synthesis of MSG.

The building blocks were also utilized to synthesize MSG, as shown below (Scheme 17). Chiral epoxide **130** was treated with acetylide anion to give homopropargylic alcohol **134**. After protection group exchange, the liberated primary alcohol **135** was oxidized to produce conjugated acid **136** after Swern/Pinnick oxidation. This carboxylic acid **136** was esterified with the previous building block **127** using Yamaguchi reagents. After PMB deprotection, free alcohol **137** was coupled with another previous building block **126** under the same conditions as for MSI (Scheme 16). Finally, RCM was carried out to synthesize the deoxygenated MS product, MSG **7**, in an 82% yield.

PTSA: p-Toluenesulfonic acid.

**Scheme 18:**
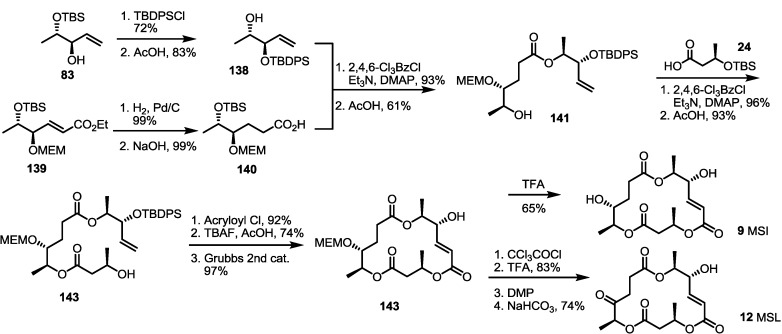
Total synthesis of MSI and L.

During the medicinal chemistry approach of MS, MSI and L were synthesized through the RCM route [47] (Scheme 18). The Nemoto group reported previously that the synthesized allylic alcohol **83** could be converted into **141** using protection group exchange and esterification with carboxylic acid **140**. After iterative esterification/deprotection, RCM was carried out to produce a dihydro-MS skeleton **143** in an excellent yield. Acidic hydrolysis of the MEM protection group produced MSI, while protection group exchange, oxidation and the final deprotection sequence produced MSL in a moderate yield.

**Scheme 19:**
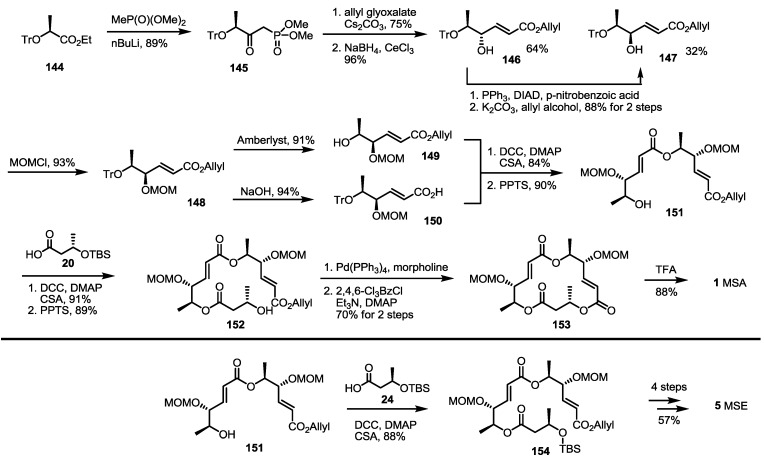
Horner–Emmons approach to MSA and E.

Traditional Wittig/Horner–Emmons olefination was also reported to be applicable for the synthesis of the MS skeleton, as shown in Scheme 19 [48]. A phosphonate synthon **147** was prepared from ethyl ester **144** [49] and the addition of methyl phosphonate in 89% yield. Horner–Emmons olefination of the phosphonate **145** with allyl glyoxalate, followed by NaBH_4_ reduction, produced allylic alcohol **146** and **147** together. Because the undesired isomer **146** was a major product, Mitsunobu inversion of the chiral center was carried out in 88% for two steps. The desired allylic alcohol **147** was then protected with the MOM group to produce bis-protected allyl ester **148** as the common substrate for further synthesis. Acidic deprotection of the trityl group produced free alcohol, **149**, which was then esterified with carboxylic acid **150**, prepared from **148** and hydrolysis, to give dimeric ester **151** after further PPTS-mediated trityl (triphenylmethyl) deprotection. The iterative esterification/deprotection sequence produced allyl ester **152** in an excellent yield. Finally, routine allyl deprotection/macrolactonization/MOM deprotection reactions produced MSA **1** in a good yield. As a coupling partner, butyric acid **24** was used to give MSE **5** after the conventional four-step sequence.

TBDPS: tert-Butyldiphenylsilyl.

**Scheme 20:**
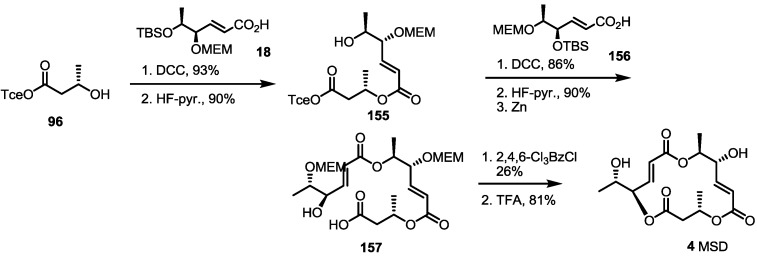
First synthesis and confirmation of the structure of MSD.

Recently, the Curran group in Pittsburgh also reported the first total synthesis and structural elucidation of MSD [50] (Scheme 20). Conventional esterification of the hydroxyl butyrate **96** with the known carboxylic acid **18** gave dimer **155** in an excellent yield after TBS deprotection. Another carboxylic acid **156** was also attached using dimer **155** under similar conditions, and the resulting product was deprotected with HF-pyridine/Zn sequentially. Finally, Yamaguchi lactonization and global deprotection produced MSD **4** in a moderate yield. It is noteworthy that the research was conducted after the fluorous mixture synthesis of MS. They synthesized all of the 16-stereoiosmer library of MS skeletons using their own fluorous tagging technique. As all of the synthetic isomers did not match with natural MSD, however, they hypothesized that MSD is a regioisomeric 15-membered lactone MS framework. After extensive NMR study, they were able to synthesize MSD by conventional methods and to revise the originally proposed structure.

**Scheme 21:**
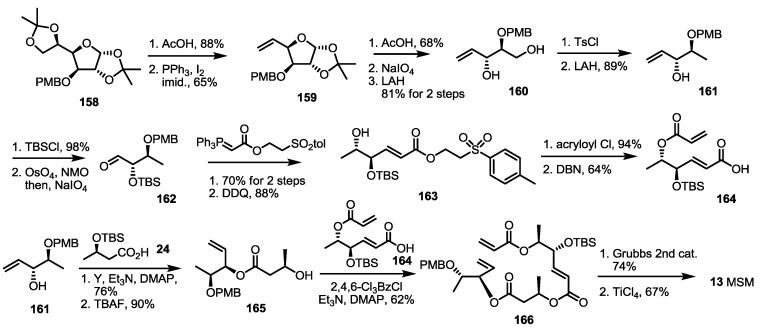
Total synthesis of MSM.

The last synthesis so far was reported by the Sharma group [51] (Scheme 21). They published a regioisomeric 15-membered lactone MSM from the sugar skeleton again. The known furanose **158** was transformed into terminal alkene **159** via hydrolysis of acetal and the elimination reaction [52]. As for the previous synthesis of MSA by the same group (Scheme 8), acidic hydrolysis/oxidative cleavage and the reduction sequence produced diol **160** in a good yield. Selective tosylation and LAH reduction was used to obtain the key building block, **161**. This allylic alcohol **161** was protected and oxidized to aldehyde **162**, which was converted into **163** via Wittig olefination/PMB deprotection sequence. Acryloylation of the secondary alcohol and basic elimination produced acryloyl acid **164** in a good yield. The allylic alcohol **161** was also used to prepare another secondary alcohol **165** via esterification with butyric acid **24** and TBS deprotection. The resulting alcohol **165** was coupled with the acryloyl acid **164** under Yamaguchi conditions to give pivotal terminal diene **166** in a moderate yield. Finally, RCM and acidic deprotection produced the 15-membered macrocycle MSM **13**.

**Scheme 22:**
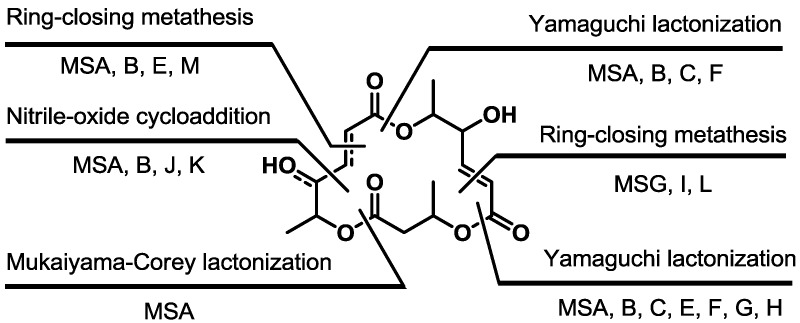
Macrocyclization of the MS skeleton.

The macrocyclization strategy is summarized in Scheme 22. This shows that various approaches have been employed to construct a unique skeleton of the macrosphelide family.

## 3. Conclusions

In conclusion, the total synthesis of MS was summarized. Efficient synthesis of monomeric fragments and macrolactonization was usually employed. For the preparation of monomers, unique synthetic methods, such as asymmetric dihydroxylation, furan oxidation, enzymatic chiral resolution, usage of sugar chirality, vinylogous orthoester addition, reduction of propargylic alcohol and Wittig olefination, were developed. For final macrolactonization, Yamaguchi-type esterification, RCM and nitrile-oxide cycloaddition have been developed so far. This synthetic study made it possible to prepare not only natural MS in a large scale, but also artificial MS with advanced pharmacological activities. Based on the accumulated results, more improved agents from MS are anticipated for the development of related molecules. The medicinal synthetic approach and more advanced synthetic research will be carried out in due course.
